# Supplementary material for: Insights into the genomic evolution of insects from cricket genomes
Source: Commun Biol. 2021 Jun 14;4:733. doi: 10.1038/s42003-021-02197-9 (PMC8203789; doi:10.1038/s42003-021-02197-9)
Supplement: Supplementary file 1 — Supplementary Information [file 42003_2021_2197_MOESM1_ESM.pdf]

Supplementary Information for

Insights into the genomic evolution of insect from Cricket genomes

Guillem Ylla, Taro Nakamura, Takehiko Itoh, Rei Kajitani, Atsushi Toyoda, Sayuri Tomonari, Tetsuya Bando, Yoshiyasu Ishimaru, Takahito Watanabe, Masao Fuketa, Yuji Matsuoka, Austen A. Barnett, Sumihare Noji, Taro Mito, Cassandra G. Extavour

This Supplementary Information file consists of the following:

- Supplementary Figures 1 - 4
- Supplementary Tables 1 - 5
- Supplementary Note 1
- Supplementary References

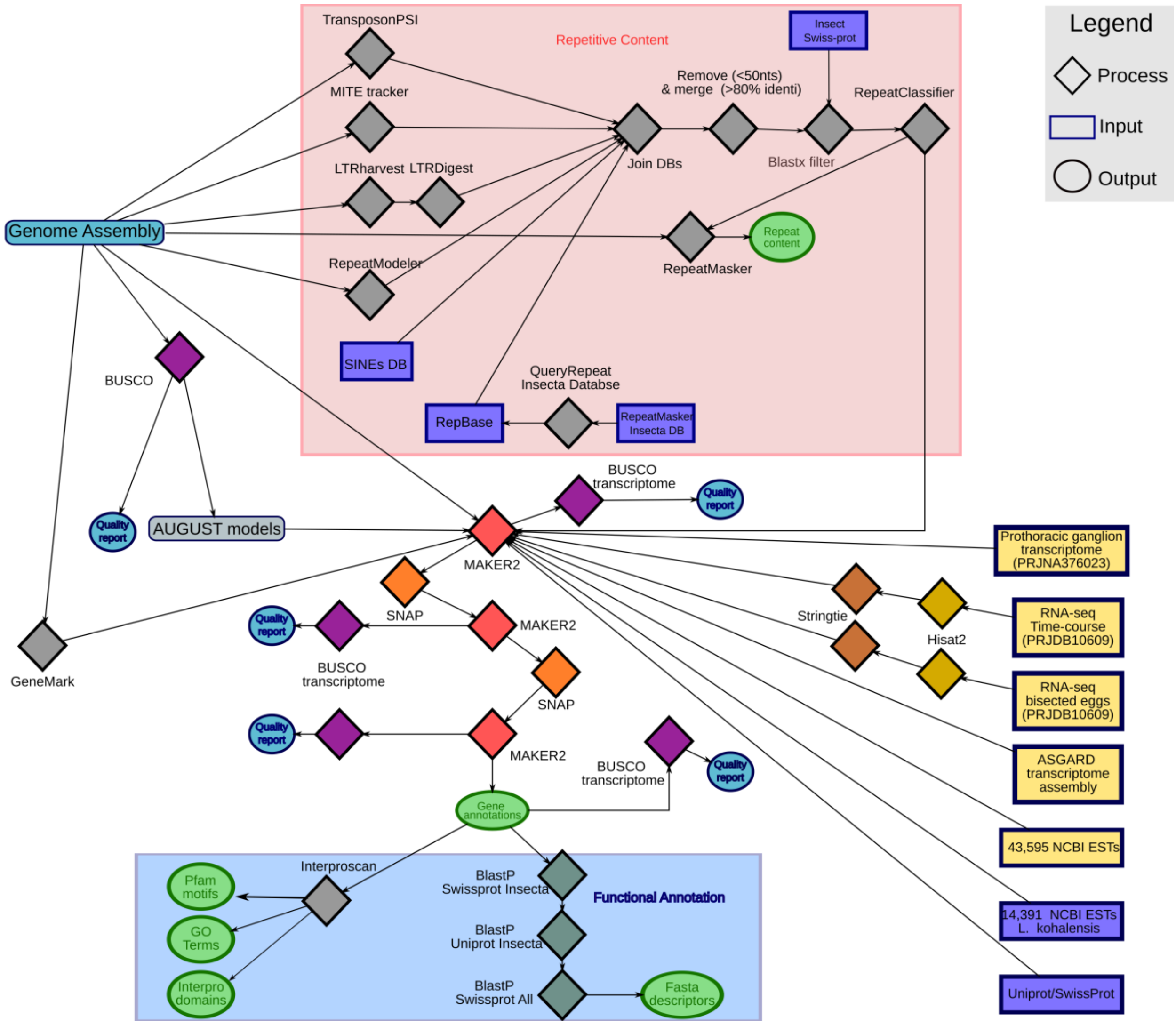

Supplementary Figure 1: Schematic of *G. bimaculatus* genome annotation pipeline. Rectangles represent data inputs: yellow rectangles represent *G. bimaculatus* data; purple rectangles represent data from other species or databases. Diamonds represent computational processes: gray diamonds indicate processes executed a single time; non-gray diamonds of the same color indicate the same process. Circles indicate outputs: blue circles indicate quality controls; green circles indicate annotations. Scripts available at GitHub https://github.com/guillemylla/Crickets_Genome_Annotation.

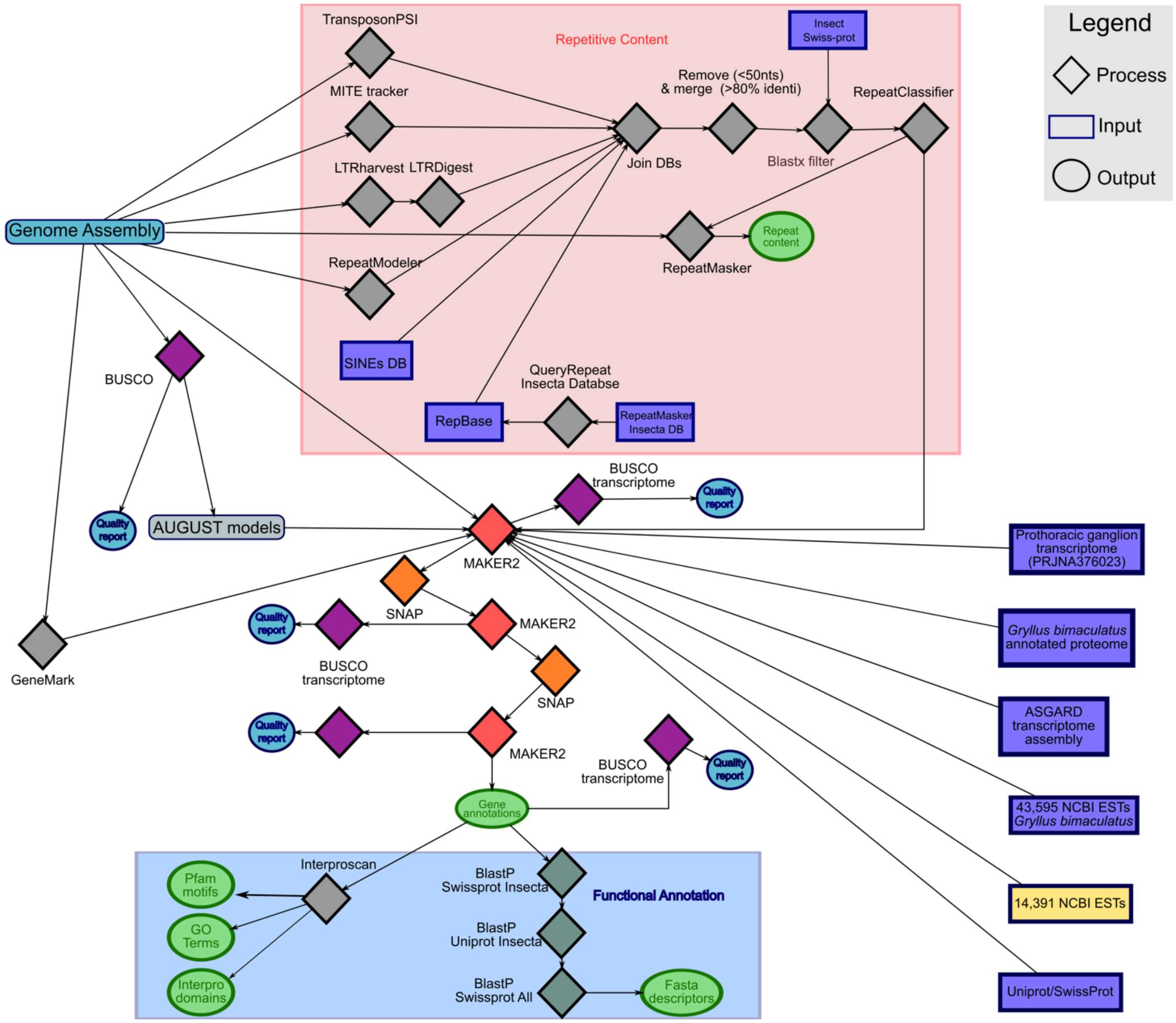

Supplementary Figure 2: Scheme of *L. kohalensis* genome annotation pipeline. All symbols as per **Supplementary Figure 1**.

40

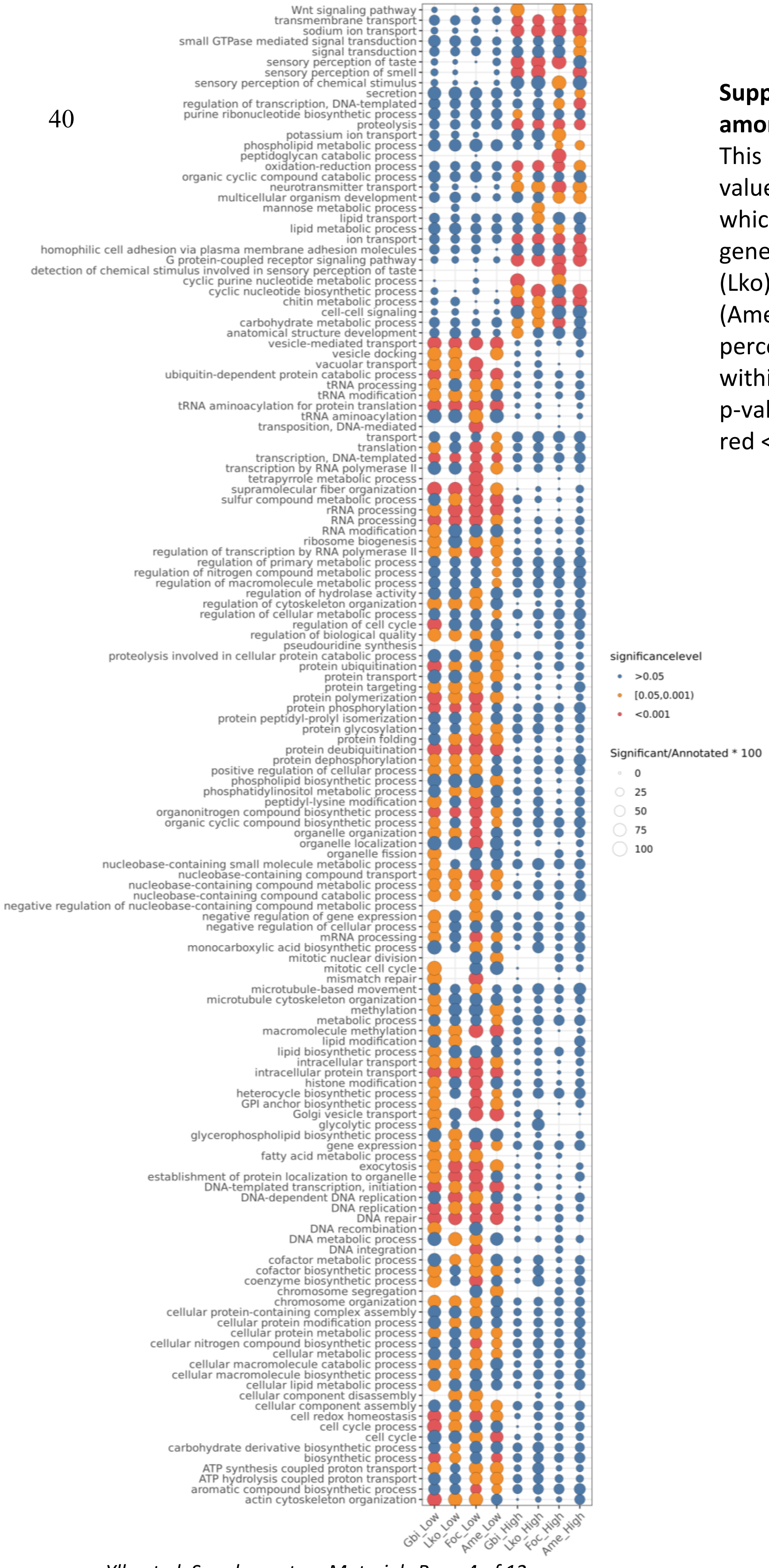

Supplementary Figure 3: Enriched GO-terms among genes with high or low CpGo/e levels. This plot shows the enriched GO terms with p-value<0.05 in at least one of the eight categories which are the high CpGo/e and low CpGo/e genes of *G. bimaculatus* (Gbi), *L. kohalensis* (Lko), *F. occidentalis* (Foc), and *A. mellifera* (Ame). The dot diameter is proportional the percentage of significant genes with the GO term within the gene set. The dot color represents the p-value level, blue >0.05, orange [0.05, 0.001), red <0.001.

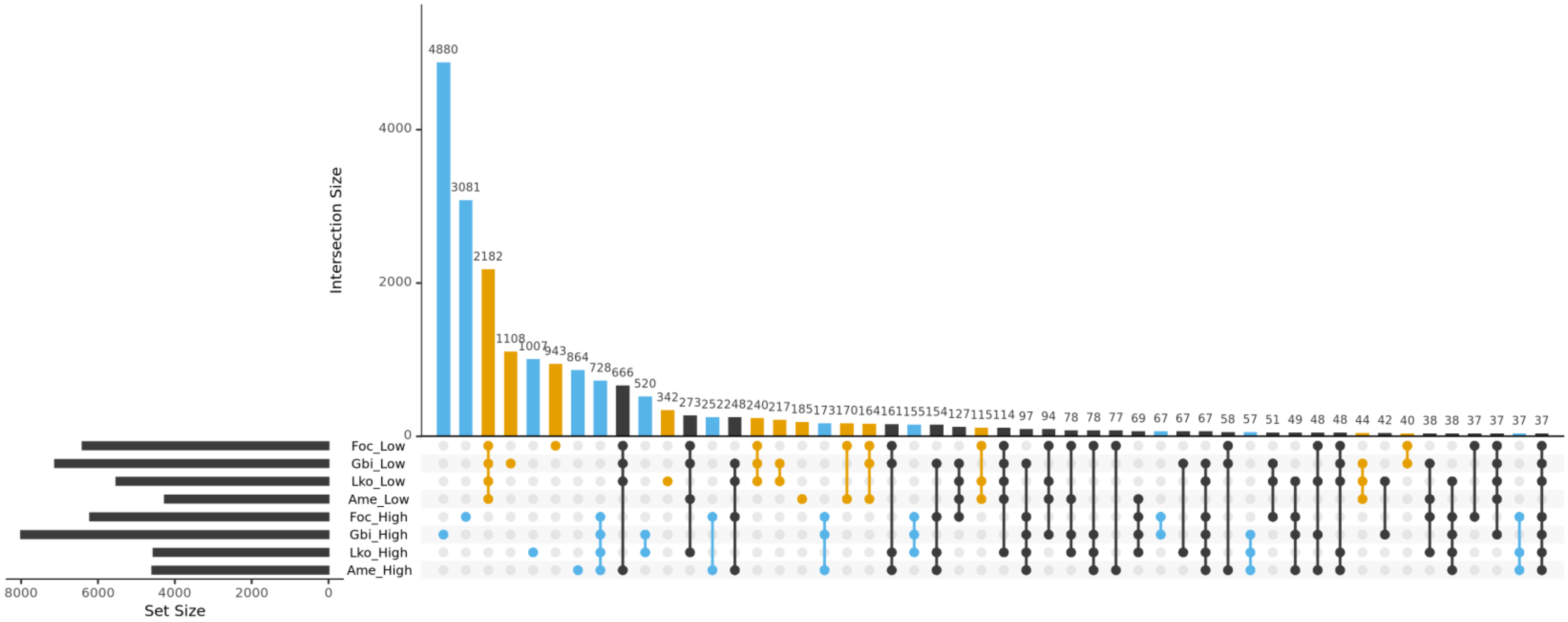

Supplementary Figure 4: UpSet plot of orthologous genes withing the high and low $CpG_{o/e}$. Top 50 intersections of orthogroups (OGs) that are common across the 8 different categories, which are the high $CpG_{o/e}$ and low $CpG_{o/e}$ genes for *G. bimaculatus* (Gbi), *L. kohalensis* (Lko), *F. Occidentalis* (Foc), and *A. mellifera* (Ame). Blue color indicates OGs that contain genes that only belong to high $CpG_{o/e}$ peak and yellow OGs that contains genes that only belong to the low $CpG_{o/e}$ and peak.

Supplementary Table 1: Number of species with available genome assembly in NCBI (RefSeq and GeneBank) for each of the 15 hemimetabolous orders and for all holometabolous species as for March 26, 2021.

Hemimetabolous Order	Number of spp with available genome assembly
Zoraptera	0
Mantodea	0
Mantophasmatodea	0
Grylloblattodea	0
Embiodea	0
Psocoptera	0
Dermaptera	1
Phthiraptera	2
Odonata	3
Ephemeroptera	3
Plecoptera	3
Thysanoptera	3
Blattodea	5
Orthoptera	6
Phasmatodea	13
Hemiptera	49

Taxon	Number of spp with available genome assembly
Holometabola	601

Supplementary Table 2: RepeatMasker summary report for *G. bimaculatus*. Report of the repeat content in the genome of *G. bimaculatus* generated by RepeatMasker using custom libraries.

Species: *Gryllus bimaculatus*
sequences: 47877
total length: 1658007496 bp (1601517380 bp excl N/X-runs)
GC level: 39.93 %
bases masked: 558652201 bp (33.69 %)

		Number of elements*	Length	Percentage
SINEs:		138895	26406967bp	1.59%
	ALUs	6	9564bp	0.00%
	MIRs	0	0bp	0.00%
LINEs:		454301	147302087bp	8.88%
	LINE1	1803	826764bp	0.05%
	LINE2	115576	32029561bp	1.93%
	L3/CR1	18286	6358119bp	0.38%
LTR elements:		131656	36970251bp	2.23%
	ERVL	92	44183bp	0.00%
	ERVL-MaLRs	0	0bp	0.00%
	ERV_classI	11451	2441461bp	0.15%
	ERV_classII	980	401749bp	0.02%
DNA elements:		500741	142828465bp	8.61%
	hAT-Charlie	11512	4094376bp	0.25%
	TcMar-Tigger	2039	537995bp	0.03%
Unclassified:		367653	126552078bp	7.63%
Total interspersed repeats:			480059848bp	28.95%
Small RNA:		2562	1002728bp	0.06%
Satellites:		31087	7528498bp	0.45%
Simple repeats:		769175	77632578bp	4.68%
Low complexity:		85129	6215377bp	0.37%

* most repeats fragmented by insertions or deletions have been counted as one element
RepeatMasker version open-4.0.5 , default mode
run with rmblastn version 2.2.27+
RepBase Update 20160829, RM database version 20160829

Supplementary Table 3: RepeatMasker summary report for *L. kohalensis*. Report of the repeat content in the genome of *L. kohalensis* generated by RepeatMasker using custom libraries.

Species*: Laupala kohalensis*
Sequences: 148784
total length: 1595214429 bp (1563778341 bp excl N/X-runs)
GC level: 35.58 %
bases masked: 566518287 bp (35.51 %)

		Number of elements*	Length	Percentage
SINEs:		29510	7083717bp	0.44%
	ALUs	304	101257bp	0.01%
	MIRs	1248	430584bp	0.03%
LINEs:		1035151	322470849bp	20.21%
	LINE1	941	367057bp	0.02%
	LINE2	584526	167380843bp	10.49%
	L3/CR1	10257	4624100bp	0.29%
LTR elements:		57347	29690552bp	1.86%
	ERVL	231	43500bp	0.00%
	ERVL-MaLRs	0	0bp	0.00%
	ERV_classI	1821	585650bp	0.04%
	ERV_classII	389	125302bp	0.01%
DNA elements:		189815	62384975bp	3.91%
	hAT-Charlie	15008	5154516bp	0.32%
	TcMar-Tigger	8896	2459752bp	0.15%
Unclassified:		409303	128822550bp	8.08%
Total interspersed repeats:			550452643bp	34.51%
Small RNA:		13816	3005585bp	0.19%
Satellites:		2088	882748bp	0.06%
Simple repeats:		307925	19782955bp	1.24%
Low complexity:		48386	2381730bp	0.15%

* most repeats fragmented by insertions or deletions have been counted as one element
RepeatMasker version open-4.0.5 , default mode
run with rmblastn version 2.2.27+
RepBase Update 20160829, RM database version 20160829

Supplementary Table 4: The orthogroups (OG) containing the 31 *D. melanogaster* pickpocket genes, with their FlyBase ID, symbol, and class according to Zelle, Lu [1].

OG	Flybase ID	Dmel symbol	Zelle 2013 class
OG0000361.fa	FBgn0034965	*ppk29*	I
OG0000361.fa	FBgn0039424	*ppk15*	I
OG0000361.fa	FBgn0051065	*ppk31*	I
OG0000361.fa	FBgn0053508	*ppk13*	I
OG0009052.fa	FBgn0032602	*ppk17*	V
OG0000185.fa	FBgn0039675	*ppk21*	III
OG0000185.fa	FBgn0039677	*ppk30*	III
OG0000185.fa	FBgn0039679	*ppk19*	III
OG0000185.fa	FBgn0065109	*ppk11*	IV
OG0000185.fa	FBgn0039676	*ppk20*	III
OG0000185.fa	FBgn0031802	*ppk7*	III
OG0000185.fa	FBgn0031803	*ppk14*	III
OG0000072.fa	FBgn0022981	*rpk / ppk2*	V
OG0000072.fa	FBgn0034730	*ppk12*	V
OG0000072.fa	FBgn0052792	*ppk8*	V
OG0000072.fa	FBgn0053289	*ppk5*	V
OG0000072.fa	FBgn0020258	*ppk / ppk1*	V
OG0000072.fa	FBgn0265001	*ppk18*	IV
OG0000072.fa	FBgn0030795	*ppk28*	V
OG0000072.fa	FBgn0035785	*ppk26*	V
OG0011276.fa	FBgn0035458	*ppk27*	IV
OG0000243.fa	FBgn0034489	*ppk6*	IV
OG0000243.fa	FBgn0039839	*ppk24*	IV
OG0000243.fa	FBgn0051105	*ppk22*	IV
OG0000243.fa	FBgn0065108	*ppk16*	IV
OG0000243.fa	FBgn0024319	*Nach / ppk4*	IV
OG0000167.fa	FBgn0050181	*ppk3*	II
OG0000167.fa	FBgn0053349	*ppk25*	II
OG0000167.fa	FBgn0065110	*ppk10*	II
OG0000167.fa	FBgn0085398	*ppk9*	II
OG0000167.fa	FBgn0030844	*ppk23*	VI

Supplementary Table 5: *pickpocket* genes present in previous QTL analyses examining the genetic basis for sound-based cricket courtship behavior variation. Genomic position information for the *L. kohalensis pickpocket* genes found in linkage groups (LG) in previously published QTL analyses[2, 3, 4] examining mating song rhythm variations and female acoustic preference in the genus *Laupala*.

								Table S3 and S6 (Blankers, Oh [2])		Table S4 (Blankers, Oh [3])	Table 2 (Xu and Shaw [4])
**Scaff names Shaw**	**Scaff Names NCBI**	**start**	**end**	**width**	**strand**	**Name**	**Ppk class**	**LG**	**proximity**	**LG**	**LG**
Lko057S000409	NNCF01126148.1	1083057	1116038	32982	+	Lko_01144	Class IV	1	LOD1	1	
Lko057S000550	NNCF01126289.1	666338	667949	1612	-	Lko_06470	Class IV	3	LOD2		
Lko057S005538	NNCF01131273.1	20948	31450	10503	-	Lko_31867	Class V	4	LOD1		
Lko057S005538	NNCF01131273.1	6676	8154	1479	-	Lko_31866	Class V	4	LOD1		
Lko057S005538	NNCF01131273.1	43198	60736	17539	-	Lko_31869	Class V	4	LOD1		
Lko057S000206	NNCF01125945.1	353321	357106	3786	-	Lko_06341	Class III				3
Lko057S000206	NNCF01125945.1	404113	432386	28274	-	Lko_06342	Class III				3

Supplementary Note 1: Genome size estimation by k-mer on a different dataset than the one used for the genome assembly. For this analysis we used 751M reads obtained from DNA of multiple specimens.

Platform	Number of Reads	Total bases	Ave. Read Length
**GAIIx**	353,824,480	52,699,721,836	148.943
**HiSeq**	398,005,266	40,198,822,874	101.001
**Total**	751,829,746	92,898,544,710	123.563

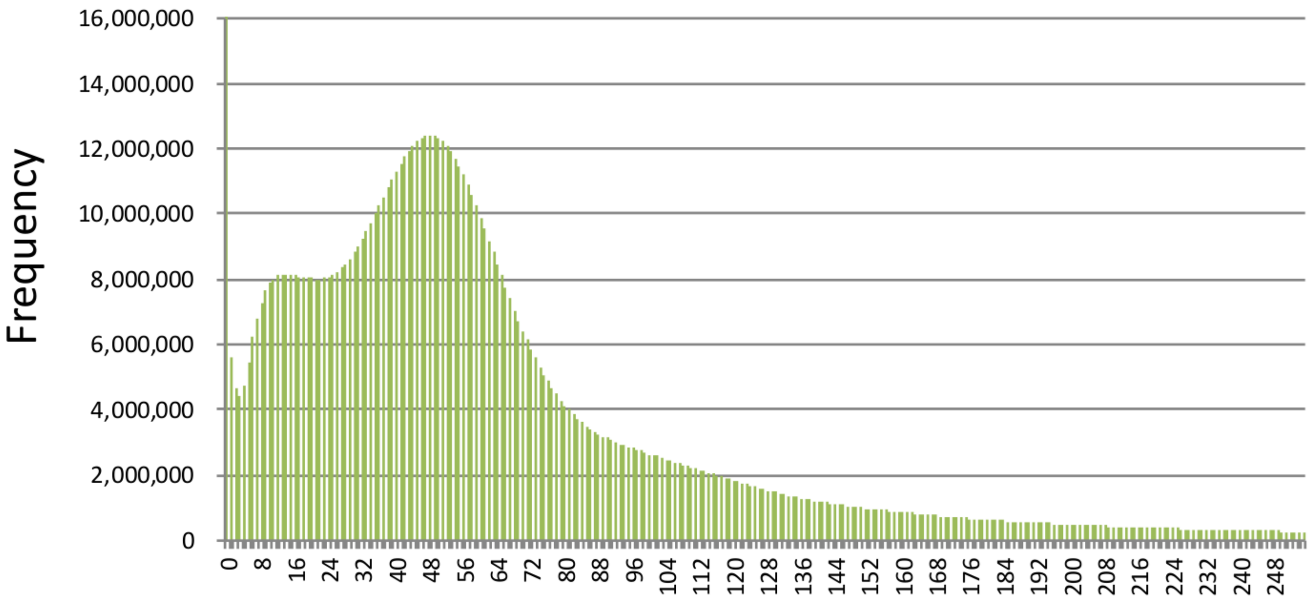

[K]	K-mer	17
[D]	Peak K-mer	48
[N]	Total Reads	751,829,746
[L]	Average Read length	123.6
[B]	Low frequency K-mer	47,170,416
[G]	Estimate Size	1,683,793,716 bp

$$[G] = \frac{\{ [N] * ([L] - [K] + 1) \} - [B]}{[D]}$$

Supplementary References

1. Zelle KM, Lu B, Pyfrom SC, Ben-Shahar Y. The genetic architecture of degenerin/epithelial sodium channels in *Drosophila*. In: *G3: Genes, Genomes, Genetics*). Genetics Society of America (2013).

2. Blankers T, Oh KP, Shaw KL. The genetics of a behavioral speciation phenotype in an Island system. In: *Genes*). Multidisciplinary Digital Publishing Institute (2018).

3. Blankers T, Oh KP, Bombarely A, Shaw KL. The genomic architecture of a rapid Island radiation: Recombination rate variation, chromosome structure, and genome assembly of the hawaiian cricket *Laupala*. In: *Genetics*). Genetics (2018).

4. Xu M, Shaw KL. The genetics of mating song evolution underlying rapid speciation: Linking quantitative variation to candidate genes for behavioral isolation. In: *Genetics*) (2019).
