# Supplementary material for: Insights into the genomic evolution of insects from cricket genomes
Source: Commun Biol. 2021 Jun 14;4:733. doi: 10.1038/s42003-021-02197-9 (PMC8203789; doi:10.1038/s42003-021-02197-9)
Supplement: Supplementary file 2 — Description of Additional Supplementary Files [file 42003_2021_2197_MOESM2_ESM.pdf]

Description of Additional Supplementary Files

Supplementary Data 1: Genome assembly information for the 16 insect genomes analyzed. For each genome, we show the database that the assembly was retrieved from, the assembly file name, the accession code, the assembly statistics obtained with assembly-stats software (https://github.com/sanger-pathogens/assembly-stats) and the BUSCO v3.1.0 reports at Arthropoda and Insecta levels.

Supplementary Data 2: Gene family expansions in crickets. Gene families (Orthogroups) significantly expanded in the lineage leading to crickets (tab 1), expanded in *G. bimaculatus* (tab 2), and expanded in *L. kohalensis* (tab 3). For each expanded orthogroup (OG), we report the expansion size as the number of genes gained, and the functional information about the OG. The functional information consists of the list of PFAMs and GO terms associated with the genes within the OG, and the list of *D. melanogaster* genes within the OG with their FlyBase summaries.

Supplementary Data 3: *pickpocket* gene expression levels in the *G. bimaculatus* prothoracic ganglion. Expression in TPMs of *fruitless* and *ppk* genes in each RNA-seq library generated from adult male prothoracic ganglia previously generated by Fisher and colleagues (2018). Genes with read sum across samples > 20 TPMs across samples are highlighted.

Supplementary Data 4: *pickpocket* gene expression levels in the *G. bimaculatus* embryo and regenerating legs. Expression in TPMs of *fruitless* and *pickpocket* genes in the aggregated embryo RNA-seq dataset, control legs, and regenerating legs. Genes with read sum across samples > 20 TPMs across samples in the prothoracic ganglion (Supplementary Data 3) are highlighted.
