# Supplementary material for: Insights into the genomic evolution of insects from cricket genomes
Source: Commun Biol. 2021 Jun 14;4:733. doi: 10.1038/s42003-021-02197-9 (PMC8203789; doi:10.1038/s42003-021-02197-9)
Supplement: Supplementary file 7 — Reporting Summary [file 42003_2021_2197_MOESM7_ESM.pdf]

nature research

Corresponding author(s): *Double-blind peer review submissions: write DBPR and your manuscript number here instead of author names.*

Last updated by author(s): *YYYY-MM-DD*

# Reporting Summary

Nature Research wishes to improve the reproducibility of the work that we publish. This form provides structure for consistency and transparency in reporting. For further information on Nature Research policies, see our Editorial Policies and the Editorial Policy Checklist.

## Statistics

For all statistical analyses, confirm that the following items are present in the figure legend, table legend, main text, or Methods section.

| n/a | Confirmed | |
|---|---|---|
| ☐ | ☒ | The exact sample size (*n*) for each experimental group/condition, given as a discrete number and unit of measurement |
| ☐ | ☒ | A statement on whether measurements were taken from distinct samples or whether the same sample was measured repeatedly |
| ☐ | ☒ | The statistical test(s) used AND whether they are one- or two-sided<br>*Only common tests should be described solely by name; describe more complex techniques in the Methods section.* |
| ☐ | ☒ | A description of all covariates tested |
| ☐ | ☒ | A description of any assumptions or corrections, such as tests of normality and adjustment for multiple comparisons |
| ☐ | ☒ | A full description of the statistical parameters including central tendency (e.g. means) or other basic estimates (e.g. regression coefficient) AND variation (e.g. standard deviation) or associated estimates of uncertainty (e.g. confidence intervals) |
| ☐ | ☒ | For null hypothesis testing, the test statistic (e.g. *F*, *t*, *r*) with confidence intervals, effect sizes, degrees of freedom and *P* value noted<br>*Give P values as exact values whenever suitable.* |
| ☒ | ☐ | For Bayesian analysis, information on the choice of priors and Markov chain Monte Carlo settings |
| ☒ | ☐ | For hierarchical and complex designs, identification of the appropriate level for tests and full reporting of outcomes |
| ☐ | ☒ | Estimates of effect sizes (e.g. Cohen's *d*, Pearson's *r*), indicating how they were calculated |

*Our web collection on statistics for biologists contains articles on many of the points above.*

## Software and code

Policy information about availability of computer code

| | |
|---|---|
| Data collection | All software used to generate the genome assembly and annotations are described in detail in the materials and methods section. In addition, the computational pipeline used for masking the repetitive content and annotating the genomes is available at https://github.com/guillemylla/Crickets_Genome_Annotation. These software are:<br>Genome assembly: Platanus (v. 1.2.1), PBjelly (v. 15.8.24).<br>Repeat annotations: LTRharvest\LTRdigest (from genometools v. 1.5.9), MITE-Tracker, TransposonPSI (v. 08222010), usearch (v. 9.2.64), RepeatClassifier, BLAST (v. 2.6.0), RepeatMasker (v. 4.0.5).<br>Genome annotations: MAKER2 (v2.31.8), AUGUSTUS (v. 3.3), BUSCO (v. 3.1.0), GeneMark-ES (v. 4.30), SNAP (v. 2013.11.29).<br>Functional annotations: InterProScan (v. 5.34-73.0), BLAST (v. 2.6.0).<br>Transcriptomic data: HISAT2 (v. 2.0.0), StringTie (v. 1.3.4). |
| Data analysis | All software used to analyze the data are described in detail in the materials and methods section. These include:<br>Genome quality analysis: BUSCO (v. 3.1.0), assembly-stats (1. 1.0.1).<br>GO enrichment analysis and visualization: TopGO, ggwordcloud, UpSetR.<br>CpGoe distribution: mixtools R package.<br>dN/dS analysis: MUSCLE (3.8.31), Pal2Nal (v. 14), PAML (v. 4.9).<br>Gene family expansions and contractions: OrthoFinder (v. 2.3.3), MUSCLE (3.8.31), GBlocks (v. 0.91b), FastTree2 (v. 2.1.11), ape, CAFE (v. 4.2.1).<br>Additional custom-made scripts are available at https://github.com/guillemylla/Crickets_Genome_Annotation/Analysis. |

For manuscripts utilizing custom algorithms or software that are central to the research but not yet described in published literature, software must be made available to editors and reviewers. We strongly encourage code deposition in a community repository (e.g. GitHub). See the Nature Research guidelines for submitting code & software for further information.

## Data

Policy information about availability of data

All manuscripts must include a data availability statement. This statement should provide the following information, where applicable:

- Accession codes, unique identifiers, or web links for publicly available datasets
- A list of figures that have associated raw data
- A description of any restrictions on data availability

The genome sequencing reads, RNA-seq reads, and the genome assembly for Gryllus bimaculatus were submitted to DDBJ and to NCBI under the accession number (PRJDB10609). The genome assembly and annotations can also be accessed and browsed at http://gbimaculatusgenome.rc.fas.harvard.edu.

# Field-specific reporting

Please select the one below that is the best fit for your research. If you are not sure, read the appropriate sections before making your selection.

☐ Life sciences ☐ Behavioural & social sciences ☒ Ecological, evolutionary & environmental sciences

For a reference copy of the document with all sections, see nature.com/documents/nr-reporting-summary-flat.pdf

# Ecological, evolutionary & environmental sciences study design

All studies must disclose on these points even when the disclosure is negative.

| | |
|---|---|
| Study description | We sequenced, assembled and annotated the Gryllus bimaculatus genome, and annotated the genome of the Hawaiian cricket Laupala kohalensis. We compared these two cricket genomes with those of 14 additional insects to obtain information regarding to the genome evolution of insects. |
| Research sample | Gryllus bimaculatus white-eyed mutant strain was reared at Tokushima University, at 29±1 ˚C and 30-50% humidity under a 10-h light, 14-h dark photoperiod. |
| Sampling strategy | Testes of a single male adult of the G. bimaculatus white-eyed mutant strain were used for DNA isolation and short-read sequencing. We used DNA from testes of an additional single individual to make a long read PacBio sequencing library for to close gaps closing in the genome assembly. |
| Data collection | TI and AT conducted sequencing by HiSeq and assembling short reads using the Platanus assembler. ST, YI, TW, MF and YM conducted DNA isolation, and gap closing of contigs. |
| Timing and spatial scale | Two male specimens were collected from the same colony. |
| Data exclusions | No data was excluded from analysis. |
| Reproducibility | All raw data is available at public databases, and bioinformatic scripts available at GitHub for reproducing the analysis. |
| Randomization | Two random male specimens were obtained from a large colony of white-eye mutants. |
| Blinding | No blinding was applied in this study, this technique does not apply to our study. |

Did the study involve field work? ☐ Yes ☒ No

# Reporting for specific materials, systems and methods

We require information from authors about some types of materials, experimental systems and methods used in many studies. Here, indicate whether each material, system or method listed is relevant to your study. If you are not sure if a list item applies to your research, read the appropriate section before selecting a response.

## Materials & experimental systems

| n/a | Involved in the study | |
|---|---|---|
| ☒ | ☐ | Antibodies |
| ☒ | ☐ | Eukaryotic cell lines |
| ☒ | ☐ | Palaeontology and archaeology |
| ☐ | ☒ | Animals and other organisms |
| ☒ | ☐ | Human research participants |
| ☒ | ☐ | Clinical data |
| ☒ | ☐ | Dual use research of concern |

## Methods

| n/a | Involved in the study | |
|---|---|---|
| ☒ | ☐ | ChIP-seq |
| ☒ | ☐ | Flow cytometry |
| ☒ | ☐ | MRI-based neuroimaging |



April 2020

## Animals and other organisms

Policy information about studies involving animals; ARRIVE guidelines recommended for reporting animal research

| | |
|---|---|
| Laboratory animals | Two Gryllus bimaculatus specimens |
| Wild animals | none |
| Field-collected samples | none |
| Ethics oversight | No ethical approval or guidance was required. |

Note that full information on the approval of the study protocol must also be provided in the manuscript.
